# In Situ Growth of COF/PVA-Carrageenan Hydrogel Using the Impregnation Method for the Purpose of Highly Sensitive Ammonia Detection

**DOI:** 10.3390/s24134324

**Published:** 2024-07-03

**Authors:** Xiyu Chen, Min Zeng, Tao Wang, Wangze Ni, Jianhua Yang, Nantao Hu, Tong Zhang, Zhi Yang

**Affiliations:** 1 National Key Laboratory of Advanced Micro and Nano Manufacture Technology, Department of Micro/Nano Electronics, School of Electronic Information and Electrical Engineering, Shanghai Jiao Tong University, Shanghai 200240, China; chenxiao00yu@sjtu.edu.cn (X.C.); wangtao_sjtu@sjtu.edu.cn (T.W.); hunantao@sjtu.edu.cn (N.H.); 2 State Key Laboratory of Integrated Optoelectronics, College of Electronic Science and Engineering, Jilin University, Changchun 130012, China; zhangtong@jlu.edu.cn

**Keywords:** covalent organic framework, hydrogel, gas sensor, ammonia, in situ growth

## Abstract

Flexible ammonia (NH_3_) gas sensors have gained increasing attention for their potential in medical diagnostics and health monitoring, as they serve as a biomarker for kidney disease. Utilizing the pre-designable and porous properties of covalent organic frameworks (COFs) is an innovative way to address the demand for high-performance NH_3_ sensing. However, COF particles frequently encounter aggregation, low conductivity, and mechanical rigidity, reducing the effectiveness of portable NH_3_ detection. To overcome these challenges, we propose a practical approach using polyvinyl alcohol-carrageenan (κPVA) as a template for in the situ growth of two-dimensional COF film and particles to produce a flexible hydrogel gas sensor (COF/κPVA). The synergistic effect of COF and κPVA enhances the gas sensing, water retention, and mechanical properties. The COF/κPVA hydrogel shows a 54.4% response to 1 ppm NH_3_ with a root mean square error of less than 5% and full recovery compared to the low response and no recovery of bare κPVA. Owing to the dual effects of the COF film and the particles anchoring the water molecules, the COF/κPVA hydrogel remained stable after 70 h in atmospheric conditions, in contrast, the bare κPVA hydrogel was completely dehydrated. Our work might pave the way for highly sensitive hydrogel gas sensors, which have intriguing applications in flexible electronic devices for gas sensing.

## 1. Introduction

Gas detection plays an essential role in environmental protection [1], human safety [2], health monitoring [3], and medical rehabilitation [4]. The use of flexible gas sensors integrated into portable electronic systems for real-time and rapid gas detection at room temperature (RT) has received increasing attention [5,6]. Intelligent gas sensors that have already been developed, such as electronic noses, electronic skin, and health bands, have been able to identify gas species and assess personal health conditions [7,8]. In particular, flexible sensors for ammonia (NH_3_) detection have been developed because it is considered a biomarker for human kidney disease [9]. High-performance sensitive materials have been designed to satisfy the demands of portable devices for NH_3_ detection [10,11,12]. For example, carbon-based nanomaterials (graphene [13,14], MXene [15], carbon nanotubes [16], etc.) within functionalized semiconductors exhibit excellent sensitivity to NH_3_ at RT. However, the aggregated deactivation of gas-sensitive nanoparticles during flexible integration limits the effectiveness of these classical sensors. At the same time, such deactivation triggers slow response times from low electron transfer rates and sensing instability caused by inferior mechanical flexibility [17]. Therefore, there is still an urgent need to design flexible sensors based on novel sensitive materials to break through the barrier of portable NH_3_ detection.

Periodic materials represented by two-dimensional covalent organic frameworks (2D COFs) with abundant nanopores and functional groups are potential candidates for gas sensors [18,19,20]. Specifically, 2D COFs can be designed to have specific interactions with target molecules by tunable functional groups and active sites. Despite these advantages, COF particles obtained using the general solvothermal synthesis method still have the risk of aggregation [21] and weak contact with electrodes [22]. This phenomenon reduces the charge carrier mobility of COF particles and is extremely detrimental to flexible sensors. In addition, the low intrinsic conductivity of most COF particles hinders progress in developing electronic devices. Recently, 2D COFs have become able to self-assemble into films on different substrates (e.g., electrodes [23], metal plates [24], or polymer films [25]) with hierarchical structures. The ordered stacking of COF film grains and tight adherence have been highlighted to address the issue of particle stacking [26]. For example, color-switching 2D COF film on indium tin oxide (ITO)-coated glass can be utilized for electrochromic stimulus-responsive materials [27]. Surface-initiated polycondensation methods can also synthesize COF coatings with superior proton conductivity, an effective route for free-standing COF films toward sensing [28]. Nevertheless, covalent rigid self-assembled COF films are not suitable for all flexible devices due to the disadvantage of elastic deformability [29]. Instead, the in situ compounding of COFs with flexible substrates by electrospinning [30], wet-chemical growth [26], and spray coating [31] have been proven to be effective means of improving the tensile properties of flexible sensors [32]. Moreover, these conductive substrates can fully address the low intrinsic conductivity barrier of COFs for electrical signal gas sensing. Above all, all-in-one COF composite sensitive materials constructed by in situ growth on flexible substrates are the rising star of high-performance flexible gas sensors.

Hydrogels serve as conductive template platforms that assemble and transfer various morphological surfaces for the manufacturing of flexible sensors in a simple and versatile manner [30]. Moreover, a mechanism based on the sensitivity of polyvinyl alcohol (PVA) hydrogels to NH_3_ has been proposed in previous work [33]. It is an opportunity for hydrogels to replace traditional silicon-based electronics for fabricating flexible wearable sensing devices. The ionization of dissolved NH_3_ introduces NH_4_^+^ and OH^−^ that can significantly improve the electrical conductivity of the PVA hydrogel [2]. As a result, hydrogel sensors are able to maintain normal conductive functions when the target gas is at a low concentration and even in the complex environment of exhaled gases. Notably, the hydrogen bonds in PVA hydrogels provide better mechanical strength contributing to the flexible integration process. However, the hydrogels inevitably display poor durability and weakened sensing performance due to water loss. Therefore, it is challenging and important to develop a flexible sensor that uses hydrogel as a flexible template combined with a sensitive COF while addressing the concern of water loss.

In this work, we innovatively use PVA-carrageenan (κPVA) as a template to grow a COF in situ to prepare a composite hydrogel gas sensor (COF/κPVA). The synergistic effect of the COF and κPVA improves gas sensing, water retention, and mechanical properties. First, a 2,5-dihydroxyterethaldehyde (DHTA) monomer was grafted onto κPVA as a nucleation site to precisely control the growth arrangement of the COF. Subsequently, ordered crystallized COF film was encapsulated on the hydrogel surface in situ by impregnation, and COF particles were generated into the conductive network. The COF/κPVA hydrogels prevent COF particles from aggregating/collapsing through a tentacle-like network of hydrogen bonds. Structural optimization enables the design of targeted charge transport pathways to achieve high-density distribution and full utilization of the active sites.

## 2. Materials and Methods

### 2.1. Materials

1,3,5-tris-(4-aminophenyl)triazine (TAPT) and DHTA were purchased from Changchun Jilin Zhongke Technology Co., Ltd. (Changchun, China), with purity ≥98%. The involved solvents included acetic acid (AcOH, Aladdin, Shanghai, China, ≥99%), tridecane (Macklin, Beijing, China, 98%), 1,4-dioxane (Aladdin, Shanghai, China, ≥99%), mesitylene (Acros organics, Geel, Belgium, 98%), and deionized water (18.2 MΩ·cm). PVA was supplied by Shanghai Chenqi Chemical Technology Co. (Shanghai, China), with an average degree of polymerization of 1750 ± 50 and 100 mesh. 1-butyl-3-vinylimidazolium bromide ([VBIm]Br, ≥99%) was purchased from the Energy Chemical (Shanghai, China) Co., Ltd. Sodium 4-vinylbenzenesulfonate (SSS, 90%) was obtained from Shanghai Macklin Biochemical Co., Ltd. (Shanghai, China). κ-Carrageenan (κCA, ≥99%) was received from Shanghai Titan Scientific Co., Ltd. (Shanghai, China). N,N′-Methylenbis-(acrylamide) (MBA) was obtained from Macklin (Shanghai, China), and ammonium persulfate (APS, 98%) was procured from Shanghai Aladdin Biochemical Technology Co., Ltd. (Shanghai, China). All chemicals were used as received.

### 2.2. Synthesis of Bare COF Film and Particles

#### 2.2.1. COF Film

The COF films were grown using a water/oil interface based on previous work [34]. In brief, TAPT and DHTA monomers were added in a 1:2 molar ratio. DHTA was dissolved in the oil phase while TAPT was in the aqueous phase with 0.2 mL AcOH (6 M) as the catalyst. After removing the undissolved monomers, the uniform acidic water and oil phases were slowly mixed in a clean container. After a reaction time of 72 h, the free-standing COF film was successfully formed at the oil/water interface. The products were washed three times in 20 mL methanol and acetone.

#### 2.2.2. COF Particles

COF particles were prepared following the solvothermal method. Typically, TAPT (21.3 mg, 0.06 mmol) and DHTA (15.1 mg, 0.09 mmol) were dissolved in 2 mL 1,4-dioxane/mesitylene mixed solvent (volume ratio: 4:1). A total of 0.2 mL AcOH (6 M) was added as the catalyst. Then, the mixture solution was sealed and heated to 120 °C for 72 h without stirring. The powder was washed three times with 20 mL THF and methanol to obtain the final products.

### 2.3. Synthesis of Bare κPVA Hydrogel

Typically, PVA (1 g) was uniformly dispersed in 12.5 mL deionized water at RT. Subsequently, κCA (0.2 g) was added and stirred at 90 °C for 2 h to form a homogeneous colloidal solution. [VBIm]Br (0.24 g), SSS (0.23 g), and MBA (1.6 mg) were separately dissolved in 1.25 mL of deionized water and added to the above solution, stirring for 1 h. Nitrogen was selected as a protective gas to exclude air from the solution. Then, 1.25 mL of APS aqueous solution (0.023 g APS) was added, and the cross-linking reaction was maintained for 2 h. After that, the κPVA hydrogel precursors with rheological properties were rapidly molded by casting molds. Following the cyclic freeze–thaw physical crosslinking method, the precursors were frozen for three cycles at −20 °C for 2 h. The hydrogels stored at 4 °C were labeled as κPVA for fabricating flexible gas sensors.

### 2.4. Synthesis of COF/κPVA Composite Hydrogels

#### 2.4.1. One-POT Method

The COF/κPVA composite hydrogel was fabricated according to the method for the fabrication of κPVA hydrogel. Distinctly, before adding the APS crosslink initiator, the TAPT (25 mg) monomer was uniformly dispersed in the colloidal solution and marked as κPVA-1. This process is designed to uniformly distribute the functional particles in the conductive network and ensure the composite hydrogel’s stability. Similarly, the hydrogel prepared by replacing the TAPT monomer with COF particles (10 mg) obtained by the solvothermal method was named κPVA-2.

#### 2.4.2. Impregnation and In Situ Growth Method

Inspired by synthesizing COF films from hydrogel immersed in the oil phase for developing COF/κPVA composite hydrogels [35], κPVA-2 containing TAPT was cut into 2 × 1 × 0.2 cm^3^ and transferred to AcOH (6 M, 2 mL) for 4 h. Subsequently, the as-obtained AcOH-swollen κPVA-2 was transferred to the oil phase containing another DHTA (1.25 mg, 2 mL) monomer grown in situ and kept for 72 h. After washing the unreacted monomers and oil, the resultant hydrogel was named κPVA-3. Alternatively, during the molding of the colloidal solution process, bare COF film was attached to the surface by self-adhesion and named κPVA-4. The bare κPVA was fully swollen in TAPT/AcOH solution to obtain a precursor with monomer and catalyst. The precursor continued in situ growth in the DHTA oil suspension, ultimately obtaining a κPVA-5 hydrogel with COF particles inside the conductive network and the COF film coated on the surface.

### 2.5. Characterization

The morphology of the obtained samples was examined by field-emission scanning electron microscope (FE-SEM, Ultra plus, Carl Zeiss, Oberkochen, Germany). Fourier transform infrared spectroscopy (FT-IR) spectra were recorded using a Thermo Fisher Nicolet 6700 (Thermo Electron Corporation, 5225 Verona Road, Madison, WI, USA) FT-IR spectrometer.

### 2.6. Sensing Performance Test of the COF/κPVA Hydrogel

An Agilent 4156 C (Santa Clara, CA, USA) was used to measure the electrical signal of the sensing performance, and the working voltage was set to 0.5 V. The COF/κPVA hydrogels were deployed into a rectangle with a size of 20 mm (length) × 10 mm (width) × 2 mm (thickness) to fabricate gas sensors. The formula of the conductivity (*σ*, S·m^−1^) was calculated using the following Equation (1) [36]:

(1) $$\sigma =\frac{d}{AR}$$

where *d* and *R* represent the effective length and resistance of the COF/κPVA composite hydrogel connected to the circuit, and *A* is the cross-sectional area of the COF/κPVA hydrogel. The response of the COF/κPVA hydrogel sensor at RT was defined as Equation (2):

Response = ∆*I*/*I*_g_ × 100% = (*I*_a_ − *I*_g_)/*I*_a_ × 100% (2)

where *I*_a_ is the current value of the gas sensor after stabilization in air, and *I*_g_ is the current value of the gas sensor after exposure to NH_3_ for a certain time. All tests were carried out in a constant atmosphere in the laboratory at a temperature of 25 °C with a relative humidity of 50 to 65%. The flow rate of the gas was regulated by mass flow controllers and the carrier gas was compressed air [37]. Two spring-loaded probes spaced 10 mm are placed vertically on the COF/κPVA hydrogels to ensure good ohmic contact between the parts.

### 2.7. Mechanical Characterization

To test the mechanical properties of the COF/κPVA hydrogels, tensile and compression tests were carried out using a tensile testing machine at RT. The tensile tests were performed using a Dynamic Mechanical Analyzer (DMAQ800, Newcastle, DE, USA) at a speed of 100 mm·min^−1^ with a 250 N load cell. The COF/κPVA hydrogels were sized into 35 mm × 5 mm × 2 mm rectangular shapes before testing. Three samples were tested for each group.

### 2.8. Water Content and Swelling Ratio Measurement

The same water content was controlled in the hydrogel to study the effect of different preparation methods on the composite hydrogel. The water content of the samples was measured by the weight change upon drying using a vacuum freeze drier. Each sample was repeated three times to ensure the results were accurate. Before swelling, hydrogels soaked in deionized water were weighed, and a tissue was used to softly remove the supererogatory water on the surfaces [38]. The water content (*C*) was calculated by Equation (3):

(3) $$C=\frac{W_{0}-W_{t}}{W_{0}}\times 100\%$$

where *W*_0_ and *W_t_* are the weights of the hydrogels before and after drying for a specific time in environmental conditions, respectively.

The freeze-dried hydrogel samples of the COF/κPVA were soaked in deionized water at 25 °C to record the swelling properties of the hydrogels. The weight of the swollen hydrogel samples was traced until the swelling equilibrium. The swelling ratio (*Q*) was calculated by Equation (4):

(4) $$Q=\frac{M_{1}-M_{0}}{M_{0}}\times 100\%$$

where *M*_0_ and *M*_1_ are the weights of the COF/κPVA hydrogels before and after soaking, respectively.

## 3. Results

### 3.1. The Design and Characterization of the COF/κPVA Hydrogel

**Figure 1:**
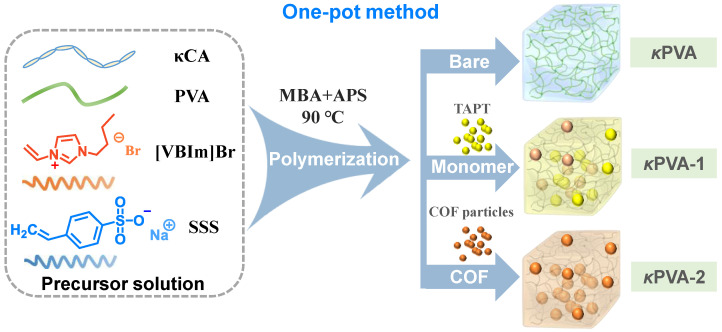
Schematic of the synthesis of bare κPVA and COF/κPVA composite hydrogels called κPVA-1 and κPVA-2 based on COF particles or the monomer using the one-pot method.

A unique flexible gas sensor for the real-time monitoring of NH_3_ can be developed by combining functionalized porous COF with the conductive κPVA hydrogel. According to the preparation process, COF-based κPVA composite hydrogels (named COF/κPVA) are divided into two types. One type of composite hydrogel is prepared using the one-pot method, with the TAPT monomer or bare COF particles directly embedded in the network during the cross-linking reaction (Figure 1). In addition, to further clearly demonstrate the composite hydrogel COF/κPVA preparation method designed in this work, the involved sensitive materials are shown in Table S1.

**Figure 2:**
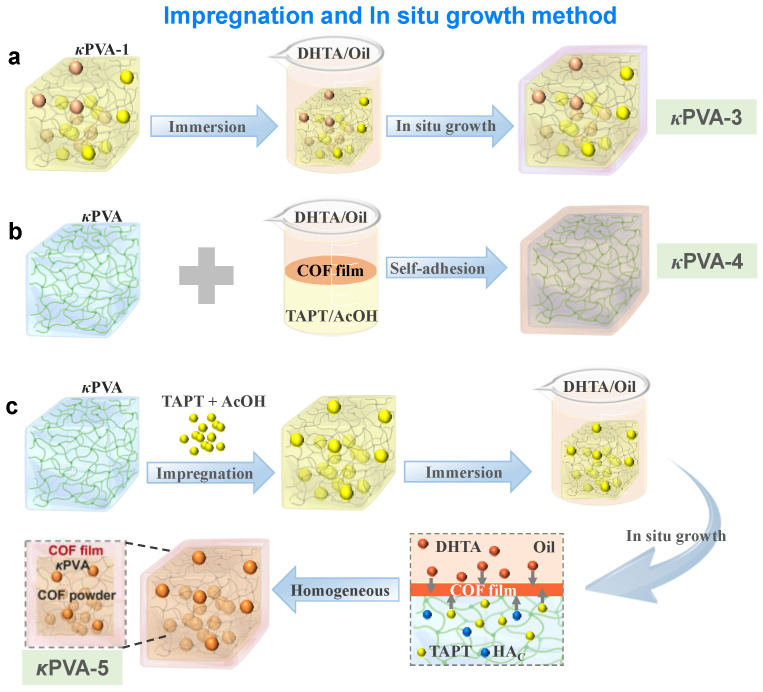
Schematic of the synthesis of COF/κPVA composite hydrogels called (**a**) κPVA-3, (**b**) κPVA-4, and (**c**) κPVA-5 using the impregnation and in situ growth methods.

The other type was based on bare κPVA, utilizing the impregnation and in situ growth processes to generate COF film/particles simultaneously on the hydrogel’s surface and inside (Figure 2c). The preparation process of functional COFs is shown in Figure S1. The functional monomer and COFs were uniformly distributed in the network and products containing unreacted monomers were avoided. In addition, the DHTA monomer can prevent the COF particles from aggregating and collapsing by constructing hydrogen bonding contacts within the network. The internal COF particles reduce energy dissipation to promote the mechanical properties of the composite hydrogels, and the COF film on the external surface improves water retention, which together extends the durability of the COF/κPVA hydrogel. Notably, the strong covalent coupling and increase in accessible active sites contributed to the high gas detection capability of the COF/κPVA gas sensors.

**Figure 3:**
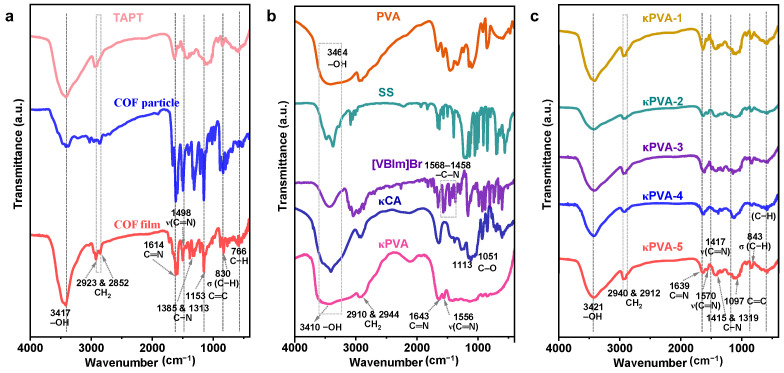
FT-IR of (**a**) bare COF particles, COF film, the monomer TAPT, (**b**) the raw materials for preparing bare κPVA, (**c**) and COF/κPVA composite hydrogels from the κPVA-1 to κPVA-5 samples.

In order to characterize the composition and microstructure of COF/κPVA hydrogels, the samples were quenched with liquid nitrogen after freeze-drying. The functional groups of COF/κPVA hydrogels were determined by FT-IR. As shown in Figure 3a, the FT-IR spectra of the bare COF particles, COF film, and the TAPT monomer are presented. The ν(C=N) stretching vibrations of COFs are located at around 1614 and 1498 cm^−1^, indicating the presence of a triazine ring [39]. However, there is no characteristic peak of TAPT at 1498 cm^−1^, which suggests that the COF has been successfully prepared by a covalent condensation reaction [40]. By comparison, the FT-IR absorption peak positions of COF film and particles are almost identical. It is confirmed that there is no difference in bonding species between COFs prepared by the interface growth and solvothermal method, which is an alternative method for large-scale preparation. The FT-IR spectra of the raw materials for the κPVA hydrogel are shown in Figure 3b. The –OH stretching vibrations of κPVA and carrageenan are located at 3464 cm^−1^ [41]. The stretching vibrations of –CH_3_ and –CH_2_ contribute to the characteristic absorption at 2944–2910 cm^−1^. Typical peaks at 1568 and 1458 cm^−1^ are ascribed to the skeleton of the imidazole ring [42]. The spectra from κPVA-1 to κPVA-5 composite hydrogels showed similar results (Figure 3c). However, the FT-IR results illustrate that there are some minor deviations from κPVA-1 to κPVA-5 depending on the state of the COFs in the composite hydrogel, including COF morphology, distribution, and COF-hydrogel binding mode (physical adhesion and in situ growth) [43,44]. It is worth mentioning that an obvious red shift occurring at –OH between the κPVA-1 to κPVA-5 sensor (3421 cm^−1^) and bare κPVA (3410 cm^−1^) can be observed, illustrating that strong hydrogen bonding is formed [45]. Meanwhile, the characteristic peaks of COFs were retained in the composite hydrogels.

**Figure 4:**
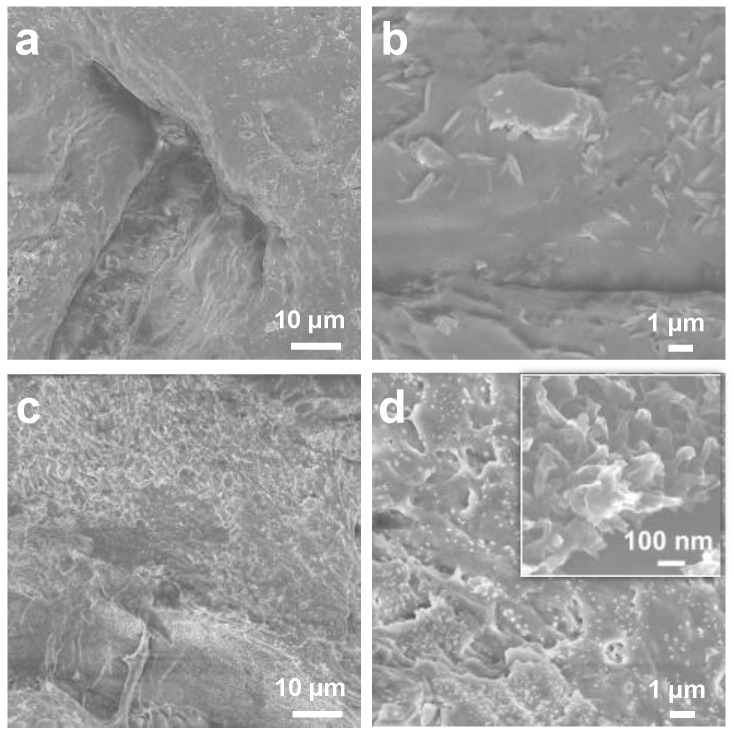
SEM images of bare κPVA with a scale bar of (**a**) 10 μm and (**b**) 1 μm. SEM images of the COF/κPVA composite (κPVA-5) with a scale bar of (**c**) 10 μm and (**d**) 1 μm (the inset image with a scale bar of 100 nm).

The microstructure of the hydrogel-based gas sensor is crucial as it can disperse the active sites of the COFs and provide channels for gas interactions [46]. As demonstrated in Figure 4a, a three-dimensional structure is formed by PVA, conducting ions, and electrostatic interactions of κPVA. The surface of κPVA hydrogel contains some minor pores and ionic crystal particles as shown in the magnification SEM image (Figure 4a,b). The κPVA-5 hydrogel formed a pore-rich spatial microstructure, which possessed similar morphologies to the κPVA hydrogel. Nevertheless, because of the COF/κPVA composite (κPVA-5) hydrogen bonding between the COF and PVA polymer chains, as shown in the inset of Figure 4d, the structure of the composite hydrogel becomes more porous in structure with the participation of the COF film. The highly porous structure of the κPVA-5 hydrogel can provide NH_3_ molecules with sufficient mobility to facilitate a rapid sensing response. The microstructural differences between these bare κPVA and composite hydrogels may also lead to different mechanical properties.

**Figure 5:**
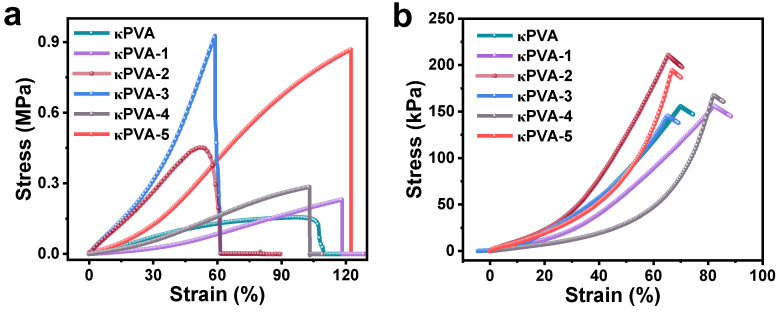
The (**a**) tensile stress−strain curves, and (**b**) compressive stress−strain curves of the hydrogel-based sensors.

To investigate whether the addition of COFs destroys the network structure of the hydrogel affecting its flexible performance, the mechanical properties were tested. The results are shown in Figure 5, and COF interactions also endow the sensors with excellent mechanical strength. Figure 5a shows the tensile stress–strain curves of sensors with different composite hydrogels. Compared to the tensile strength of bare κPVA of 0.16 MPa, κPVA-1 and κPVA-2 showed a significant improvement in tensile strength caused by the particles. The tensile strength of κPVA-3 increased dramatically to 0.92 MPa, which could be attributed to the TAPT reacting with the hydrogel and changing the elasticity. However, this reaction destroyed the κPVA-3 hydrogel network structure with a breaking strain of only 60%. With the combination of the impregnation and in situ growth methods, κPVA-5 had a tensile strength of 0.87 MPa, and tensile deformation could reach 120%. At the same time, the compressive stress–strain curves are shown in Figure 5b. Similar to the tensile properties of the sensors, the compressive strength of the κPVA-5 sensor reached 194.4 kPa at 66.5% strain. The tight adherence of the κPVA hydrogel chains to the COF film/particles by tentacle-like hydrogen bonding maximizes the mechanical strength of κPVA-5.

### 3.2. Gas Sensing Properties of the Hydrogel-Based Devices

**Figure 6:**
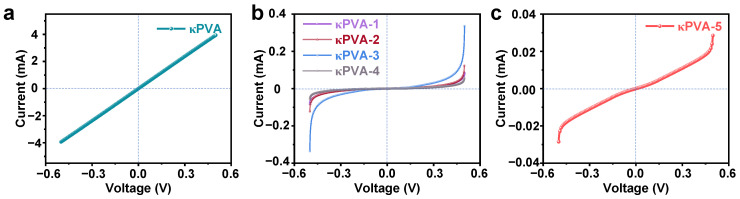
*I*–*V* curves of (**a**) bare κPVA, (**b**) κPVA-1~κPVA-4, and (**c**) κPVA-5 composite hydrogels-based sensors under ±0.5 V bias at RT.

The conductivity of the hydrogels is a prerequisite for their use as chemiresistive gas sensors. The combination of the *I*–*V* curve and conductivity equation was used to evaluate the electrical properties of the COF/κPVA composite hydrogels. The results presented in Figure 6 suggest that bare κPVA showed the optimal conductivity of 3.91 S·m^−1^. The conducting capacity of COF/κPVA hydrogels decreased with the incorporation of COF, which has a lower intrinsic conductivity [47]. The κPVA-1~κPVA-5 conductivities were 0.159, 0.158, 0.275, 0.0875, and 0.0191 S·m^−1^, respectively. In addition, the spring-loaded probes would contact the COF film expending on the hydrogels, causing a significant decrease in the conductivity of κPVA-4 and κPVA-5.

**Figure 7:**
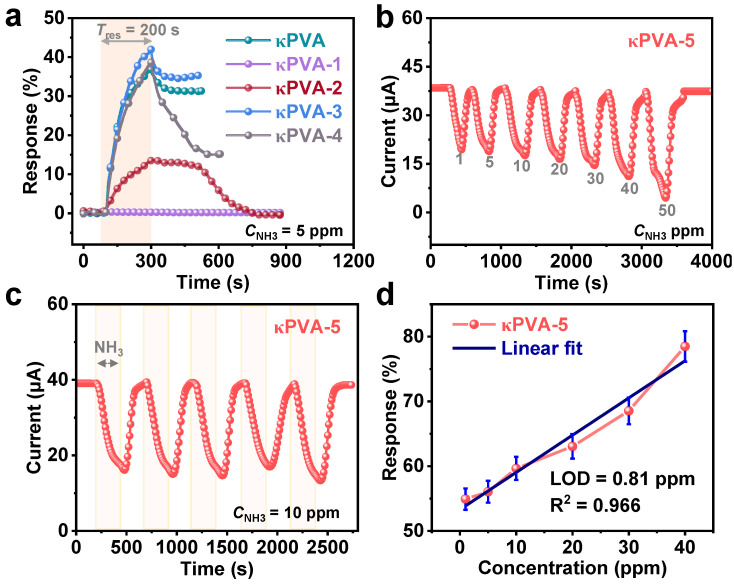
(**a**) Dynamic response curve of bare κPVA and κPVA-1~κPVA-4 hydrogel-based sensors exposed to 5 ppm NH_3_. (**b**) Dynamic current curve of the κPVA-5 hydrogel-based sensor exposed to different concentrations of NH_3_ (1, 5, 10, 20, 30, 40, and 50 ppm). (**c**) The current curve of the κPVA-5 hydrogel sensor to 10 ppm NH_3_ for five cycles. (**d**) The linear fitting curve of the κPVA-5 hydrogel-based sensor responses to NH_3_ gas with different concentrations.

The sensing capability of the self-responsive COF/κPVA hydrogels is evaluated by recording their current variation upon exposure to the NH_3_ at RT. The hydrogels were tested in the homemade detection chamber presented in Figure S2 and stabilized for 300 s before each test. The exposure of the sensors to the target gas was maintained for 200 s while venting compressed air to assist in recovery. The COF film and powder exhibited no response signal to NH_3_ (Figure S3). Except for the κPVA-2 hydrogel containing COF particles, which obtained a complete response and recovery curve to NH_3_, the rest of the samples (bare κPVA, κPVA-3, and κPVA-4 hydrogels) failed to recover (Figure 7a). What is worse, the κPVA-1 hydrogel did not respond to NH_3_, and we hypothesized that the unstable TAPT monomer destroyed the κPVA network structure by reaction with the added raw materials. Remarkably, the sensing performance of the κPVA-5 hydrogel in Figure 7b exhibited the highest sensitivity in detecting NH_3_. When exposed to the same 5 ppm NH_3_ atmosphere as the other samples, the κPVA-5 hydrogel achieved 56.1% response values and a full recovery time of 93 s (root mean square (RMS) error of less than 5%). This is attributed to the COF film with abundant porous and active sites acting as the NH_3_ trap, while the uniformly distributed COF particles constitute good charge transport pathways in the hydrogel network of κPVA-5. Selectivity tests of κPVA-5 in different atmospheres also showed specificity for NH_3_ (Figure S4). The response/recovery curve of the κPVA-5 hydrogel sensor exposed to 10 ppm NH_3_ was monitored for five cycles and, as expected, it was stable (Figure 7c). The response value also showed a gradient change when exposed to different concentrations of NH_3_. A relatively highly linear relationship was exhibited within the measurement interval, with a variance value of 0.966 for the linear fit curve (Figure 7d).

**Figure 8:**
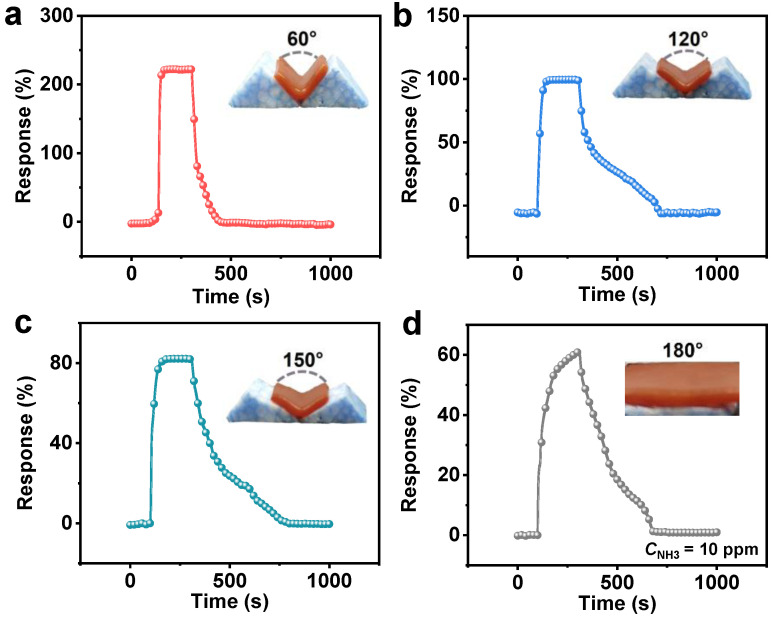
Dynamic response curves of κPVA-5 hydrogel-based sensor to 10 ppm NH_3_ at bending angles of (**a**) 60°, (**b**) 120°, (**c**) 150°, and (**d**) 180°.

A flexible self-responsive hydrogel sensor has the potential to be a wearable device that is attached to human skin for monitoring. Therefore, it is necessary to investigate the NH_3_ detection capability of the hydrogel under different bending angles (Figure 8). The hydrogel was flatly placed on a fixed-angle base mold, ensuring that the spring-loaded probes lightly touched the bend center. The response values and recovery times of the κPVA-5 hydrogel at each angle have been shown in Table S2. The results demonstrated a higher response value and quicker recovery of the κPVA-5 hydrogel sensor from 180° (flat state) to bending at 60°. Especially in the case of bending at 60°, the response value can be as high as 222%, and the recovery time significantly reduced to 104 s. In addition, under the 180° test condition, the response value of the κPVA-5 hydrogel could not reach equilibrium within the 200 s exposure time. This may be due to the shorter electron transfer distance [48] in the hydrogel network under the bending condition and rapidly achieving the saturation adsorption of the gas molecules.

### 3.3. Water Content, Swelling Ratio, and Water Retention Capacity of Hydrogels

**Figure 9:**
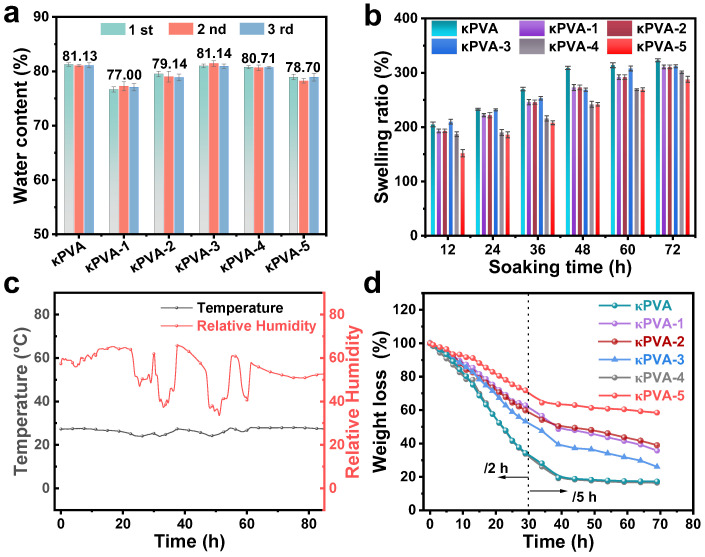
(**a**) Water content (repeated three times), and (**b**) swelling ratios in 72 h of the COF/κPVA hydrogel sensors. (**c**) Dynamic monitoring curves of temperature and relative humidity of the experimental atmosphere. (**d**) Weight changes of hydrogels in each period (2 h intervals for the first 30 h and at 5 h intervals for the next 40 h).

One of the key factors limiting the practical application of hydrogels is their instability. The flexibility of hydrogels is derived from their abundant water molecules, which makes the water content, swelling ratio, and water retention capacity of the samples important [49]. The water content of the bare κPVA hydrogel was 81.13% (RMS error of less than 3%) based on the average of three measurements (Figure 9a). Among the hydrogels, the water content of κPVA-3 might have increased due to the reaction of the monomer with the conductive ions allowing the ion exchange enhancement during the soaking process. The other composite hydrogels showed a slight decrease owing to the incorporation of COF particles and film. The swelling ratios of the hydrogel sensors were also explored and illustrated in Figure 9b. The COF dispersed within the conductive network would sacrifice a part of the swelling ratio, but mechanical stability simultaneously is highly improved [50]. Moreover, the COF film generated on the surface of the composite hydrogel fully contributes to the water retention capacity. The water loss of hydrogels observed in an atmospheric environment is displayed in Figure 9c. Because the weight of hydrogel changes rapidly in the initial period, the data were collected at 2 h intervals for the first 30 h and at 5 h intervals for the next 40 h. The κPVA-based sensor could no longer maintain operation after 40 h with only 19.6% of the weight remaining. In contrast, κPVA-5 showed the best water retention ability and a stable weight loss of 58.4% at 70 h. This is attributed to the dual effects of the COF film protection on the outside and the internal COF particles anchoring the water molecules through hydrogen bonding.

In addition, we attempted to light a bulb by connecting the water-loss hydrogels into a circuit. As shown in Figure S5, the bare κPVA in the circuit could not brighten a bulb at 20 V, and the hydrogel was in a stiff state. Instead, the κPVA-5 composite hydrogel only required 3 V to light the bulb, and the physical picture of κPVA-5 shows that it can still be bent after 70 h. A comparison of the performance of this work with others that have been reported on detecting techniques for NH_3_, with particular emphasis on response values, detection methods, and stability, is summarized in Table S3 [51,52,53,54,55,56,57]. Generally, some of these efforts faced the challenges of baseline drift and incomplete recovery. In contrast, the COF/κPVA composite hydrogel prepared in this work constitutes a stable sensor with excellent gas-sensing performance and inspires the preparation of high-performance flexible NH_3_ sensors.

### 3.4. Sensing Mechanism of the Hydrogel-Based Sensors

**Figure 10:**
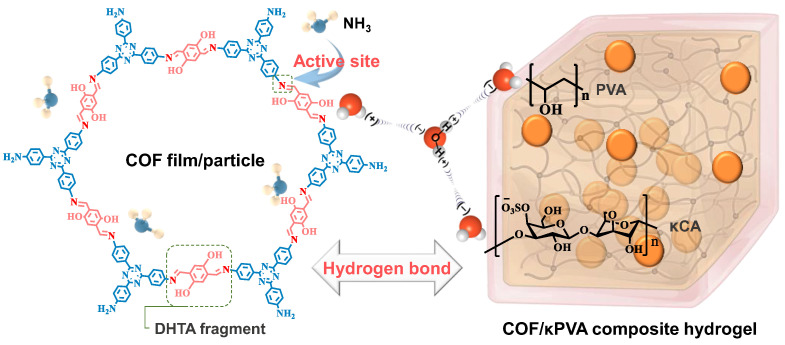
Schematic illustration of the sensing mechanism by the COF/κPVA composite hydrogel (represented by the optimal performance sample, κPVA-5) sensor for NH_3_.

The abundant –OH and imine N in the COF structure are the active sites, and the porous morphology facilitates gas molecular transport and charge transfer [58,59,60,61]. Regarding the interior of the hydrogel containing COF particles, the sensing mechanism is also different from conventional semiconductor sensing materials (Figure 10). The hydrogen bond formed between the NH_3_ molecule and –OH/–NH_2_/–SO_3_ on the conductive network triggers a variation in electrical signals for sensing. Benefiting from the hydroxyl group in the COF topology, it can not only capture NH_3_ but also become a gripper for free water molecules in the hydrogels. As a result, it enhances the ability to detect NH_3_ and extends the hydrogel sensor’s lifetime with a high water retention capacity. In addition, unlike other functional materials, COF is a porous framework with a topological structure. This means that an ordered tentacle-like array of hydrogen bonds can be obtained by in situ growth in κPVA, preventing COF particles from aggregating/collapsing like traditional materials. Consequently, the COF and PVA synergistic impact improves the sensing, water retention, and durability of the composite hydrogels.

## 4. Conclusions

In summary, we have demonstrated a practical approach to fabricating highly responsive hydrogel gas sensors using COFs integrated into PVA hydrogel templates. Our study achieves remarkable performance improvements: The COF/κPVA hydrogel exhibits an impressive 54.4% response to just 1 ppm NH_3_ (RMS error of less than 5%), with a rapid and full recovery. This represents a significant advancement over the low response and non-recovery observed with bare κPVA sensors. Furthermore, the COF/κPVA hydrogel remains stable after exposure to atmospheric conditions for 70 h. In stark contrast, the bare κPVA hydrogel is completely dehydrated under similar conditions, underlining the superior water retention properties of our composite sensor. Even under severe 60° bending, the COF/κPVA sensor maintains a high response value, reaching up to 222% when exposed to 10 ppm NH_3_. This enhanced mechanical flexibility is crucial for applications in wearable and flexible electronic devices, where durability is a key factor. These quantitative results underscore the remarkable performance enhancements achieved by our COF/κPVA composite. Beyond advancing the field of gas sensing, our research holds substantial promise for improving medical diagnostics, environmental monitoring, and various other applications where accurate and stable NH_3_ detection is vital.

## Data Availability

Data are contained within the article and Supplementary Materials.

## Supplementary Materials

The following supporting information can be downloaded at: https://www.mdpi.com/article/10.3390/s24134324/s1, Figure S1: Schematic illustration of the preparation process of functional COF film and particles; Figure S2: Schematic image of the gas testing chamber; Figure S3: The dynamic response curves of bare (a) COF film and (b) particles to the 5 ppm NH_3_; Figure S4: Responses of the κPVA-5 sensor to 10 ppm other analytes; Figure S5: Brightness images of small bulbs in circuits after (a) bare κPVA and (b) κPVA-5 hydrogels have lost water for 70 h. (c) Physical pictures of bare κPVA and (d) κPVA-5 after loss of water; Table S1: Method and name of the sensitive materials involved in this work; Table S2: The response value and recovery time of κPVA-5 hydrogel sensor to 10 ppm NH_3_ at different bending angles; Table S3. Comparisons of NH_3_ detection performance between COF/κPVA and other materials-based gas sensors.
