# Supplementary material for: In Situ Growth of COF/PVA-Carrageenan Hydrogel Using the Impregnation Method for the Purpose of Highly Sensitive Ammonia Detection
Source: Sensors (Basel). 2024 Jul 3;24(13):4324. doi: 10.3390/s24134324 (PMC11244185; doi:10.3390/s24134324)
Supplement: Supplementary file 1 [file sensors-24-04324-s001.zip › sensors-2996119-supplementary.pdf]

Supporting information for

In Situ Growth of COF/PVA-Carrageenan Hydrogel Using the Impregnation Method for the Purpose of Highly Sensitive Ammonia Detection

Xiyu Chen [1], Min Zeng [1,*], Tao Wang [1], Wangze Ni [1], Jianhua Yang [1], Nantao Hu [1], Tong Zhang [2], and Zhi Yang [1,*]

[1] National Key Laboratory of Advanced Micro and Nano Manufacture Technology, Department of Micro/Nano Electronics, School of Electronic Information and Electrical Engineering, Shanghai Jiao Tong University, Shanghai 200240, China; chenxiao00yu@sjtu.edu.cn (X.C.); wangtao_sjtu@sjtu.edu.cn (T.W.); hunantao@sjtu.edu.cn (N.H.)

[2] State Key Laboratory of Integrated Optoelectronics, College of Electronic Science and Engineering, Jilin University, Changchun 130012, China; zhangtong@jlu.edu.cn

* Correspondence: minzeng@sjtu.edu.cn (M.Z.); zhiyang@sjtu.edu.cn (Z.Y.)

Supporting Figures and Tables

The reason for the varying FT-IR results depends on the state of COF in the composite hydrogel κPVA-1 to κPVA-5, including COF morphology (particles & film), content and distribution (internal & external), COF and hydrogel binding mode (physical adhesion & in situ growth).

In Figure 3(c), for the C=N peak at 1570 cm^{-1} in κPVA-5, due to the torsional vibration of the structure triggered by the triazine bonds in the COF. κPVA-5 has a COF film encapsulated on the outside and nested COF particles on the inside, with a uniform distribution and high content of COF, so the C=N peak is obvious. In contrast, the κPVA-3 and κPVA-4 have low and inhomogeneous COF content. This is the main reason for the absence of 1570 cm^{-1} in FT-IR spectra of κPVA-3 and κPVA-4. However, for κPVA-3, the TAPT monomer powder was added to the hydrogel precursor solution, and an adverse reaction may have occurred during the polymerization reaction. Since monomeric TAPT is present in the composite hydrogel component, the peak position marked by the gray dashed line 1417 cm^{-1} does not appear in κPVA-2 and κPVA-4/5, while appearing in κPVA-1 and κPVA-3.

In addition, κPVA-4 was prepared by physically adhering a COF film to the hydrogel surface, with no internal COF inside at all, which is completely different from that obtained by the in situ growth method. According to the study, the broad peak at 1300–1500 cm^{-1} corresponds to the oxygen-hydrogen bending vibration [41,42]. It was hypothesized that peaks 1415 and 1319 cm^{-1} attributed to this range proved that the COF was hydrogen bonded to the hydrogel, thus it was not present in κPVA-4 which physically adhered to the COF and was present in all other samples.

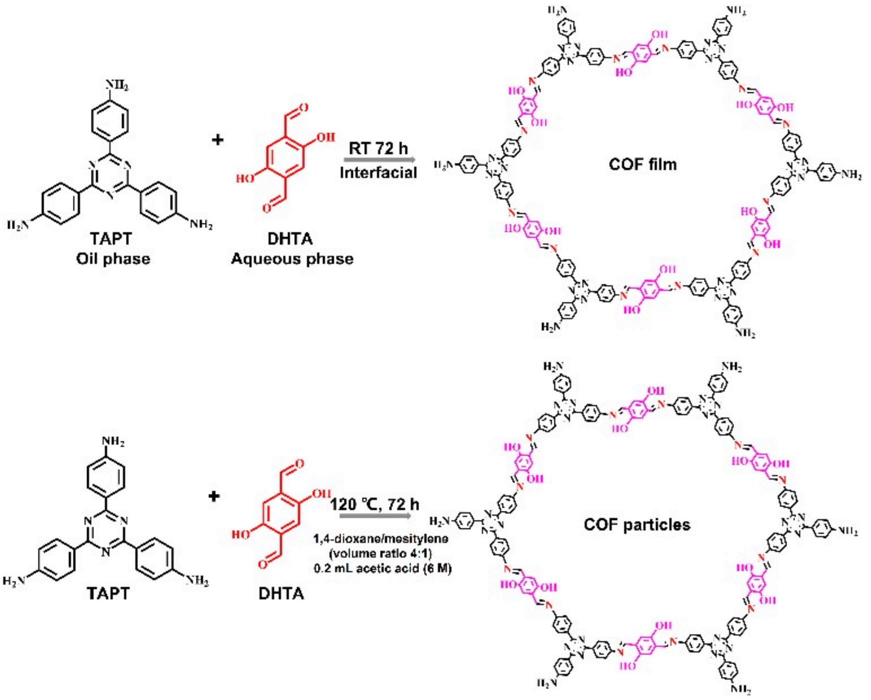

Figure S1. Schematic illustration of the preparation process of functional COF film and particles.

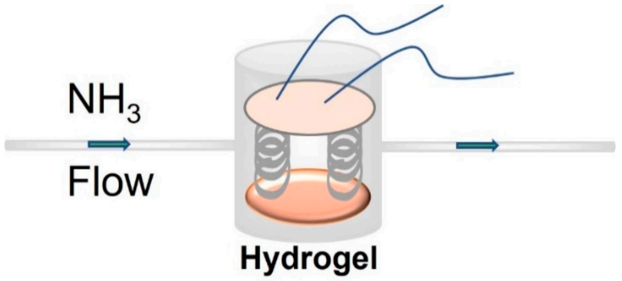

Figure S2. Schematic image of the gas testing chamber.

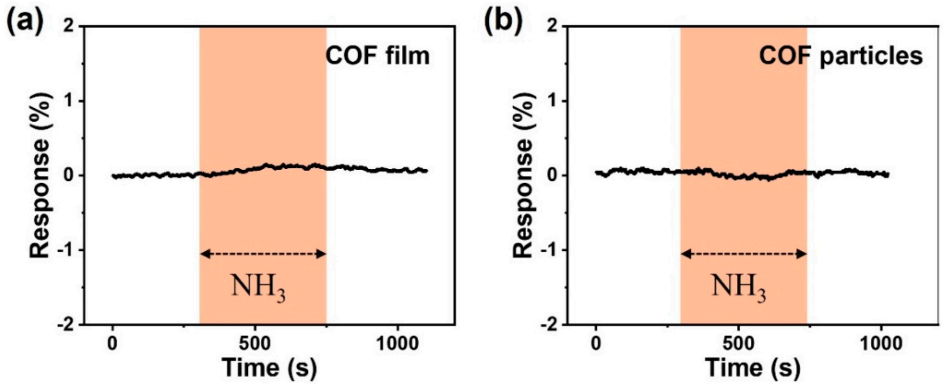

Figure S3. The dynamic response curves of bare (**a**) COF film and (**b**) particles to 5 ppm NH_3.

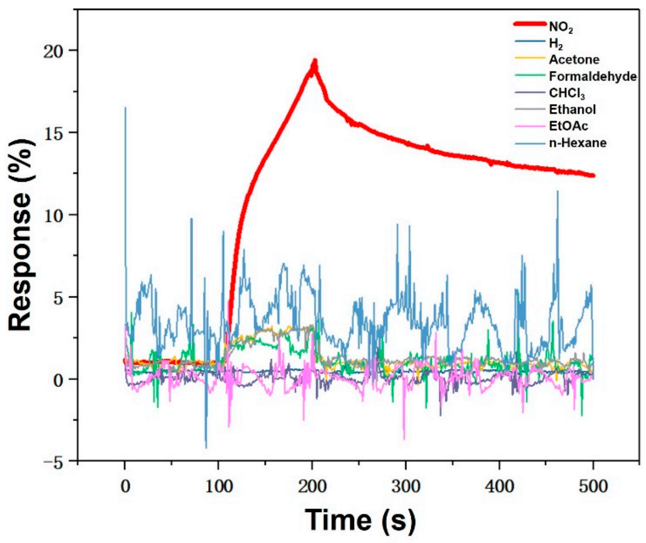

Figure S4. Responses of the κPVA-5 sensor to 10 ppm other analytes.

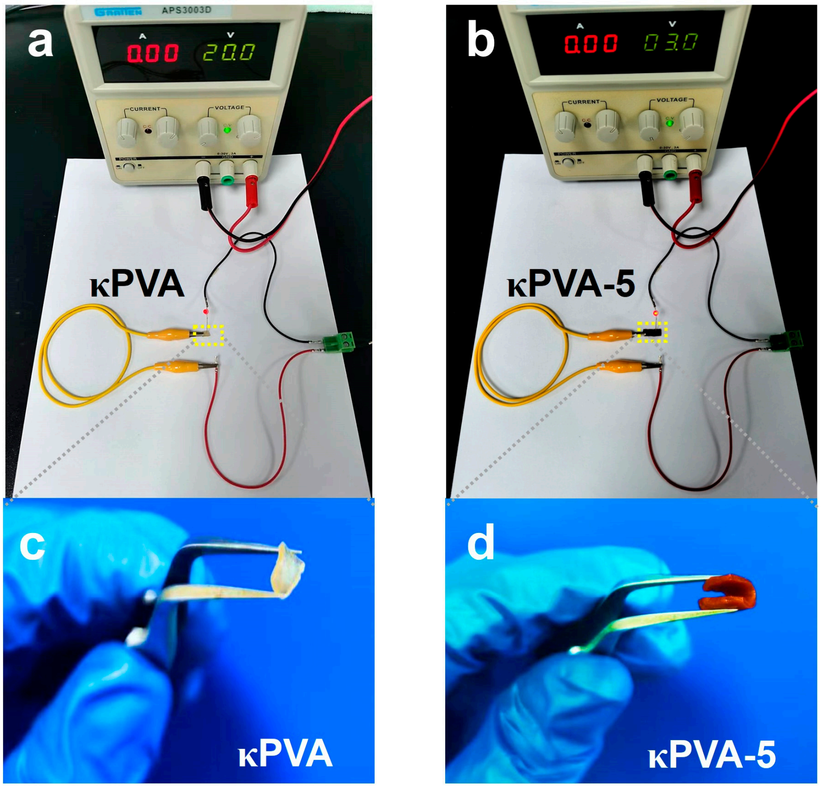

Figure S5. Brightness images of small bulbs in circuits after (**a**) bare κPVA and (**b**) κPVA-5 hydrogels have lost water for 70 h. (**c**) Physical pictures of bare κPVA and (**d**) κPVA-5 after loss of water.

Table S1. Method and name of the sensitive materials involved in this work.

Material types	Named	Raw materials	Methods	Composition	NH_3 detection
**Bare**	COF film	TAPT, DHTA monomers	Water/oil interface	COF film	×
	COF particles	TAPT, DHTA monomers	Solvothermal	COF particles	×
	κPVA	κCA, PVA, [VBIm]Br, SSS, MBA, APS	Crosslinking	κPVA	⍻ No recovery
**Composite hydrogel COF/κPVA**	κPVA-1 hydrogel	κCA, PVA, [VBIm]Br, SSS, MBA, APS + TAPT monomer	One-pot method	TAPT monomer, COF particles, κPVA	×
	κPVA-2	κCA, PVA, [VBIm]Br, SSS, MBA, APS + COF particles	One-pot method	COF particles, κPVA	√ Low response value
	κPVA-3	κPVA-1 + DHTA/Oil	In situ growth	TAPT monomer, COF particles, COF film, κPVA	⍻ No recovery
	κPVA-4	κPVA + COF film	Physical adhesion	COF film, κPVA	⍻ No recovery
	κPVA-5	κPVA + TAPT/AcOH + DHTA/Oil	Impregnation & in situ growth	COF particles, COF film, κPVA	√ Optimized performance

Table S2. The response value and recovery time of κPVA-5 hydrogel sensor to 10 ppm NH_3 at different bending angles.

Bending angle	Response value (%)	Recovery time (s)
60°	222	104
120°	99	359
150°	82	397
180° (flat state)	61	354

Table S3. Comparisons of NH_3 detection performance between COF/κPVA and other materials-based gas sensors.

**Sensing materials**	**Response value (%)**	***C* (ppm)**	**Response/ Recovery time (s)**	**Gases**	**Methods**	**Stability**	**Refs.**
P3HT/PS	52/16	50/5	NA	NH_3	OFET	Baseline drift	[49]
MoS_2/P3HT (0.5 mL)	≈ 13	20	200/> 550	NH_3	OTFTs	Baseline drift	[50]
3D S-RGO hydrogel	7.1	20	16/> 900	NH_3/NO_2	IDEs with Pt microheater	Incomplete recovery	[51]
PGA/GA hydrogels	89.2	50	NA	NH_3	ceramic plate + QCM	No response (RT)/Stabilization (High RH)	[52]
PVA hydrogel	31.6	40	t_{50} = 46/t_{50} = 29	NH_3/NO_2	Self-responsive	Baseline drift	[31]
steamed GO hydrogel	≈ 1	200 ppm	NA	NH_3/NO_2	Au IDEs	Baseline drift	[53]
TAPB-PDA-COFs	6	500 ppm	5 ± 2/6 ± 2	NH_3	Ag/AgCl electrodes	Stabilization	[54]
COF/κPVA	61	10 ppm	200/354	NH_3	Self-responsive	Stabilization	This work
